# Comparing the profile of patients with anorectal malformations and delayed presentation of anorectal malformations in the Johannesburg paediatric colorectal clinic: a retrospective study

**DOI:** 10.1007/s00383-025-06072-0

**Published:** 2025-06-24

**Authors:** A. A. Piperidis, J. Scribante, C. Bebington, G. Brisighelli

**Affiliations:** 1 Department of Paediatric Surgery, School of Clinical Medicine, Faculty of Health Sciences, University of the Witwatersrand, Johannesburg, South Africa; 2 Johannesburg Paediatric Colorectal Clinic, Chris Hani Baragwanath Academic Hospital, Johannesburg, South Africa; 3 Surgeons for Little Lives, Johannesburg, South Africa

**Keywords:** Anorectal malformations, Delayed diagnosis, Complication, Lower- and middle-income country

## Abstract

**Purpose:**

Delayed presentation of anorectal malformations (ARM) presents clinical challenges, leading to complications and compromised outcomes. This study aimed to compare the profiles and outcomes of patients with delayed presentation of ARM at the Johannesburg Paediatric Colorectal Clinic (JPCC).

**Methods:**

A retrospective review of patients seen at JPCC between 2018 and 2023 was conducted. Patients diagnosed after 24 h from birth were considered delayed. (Ethics approval M240346).

**Results:**

All 269 ARM patients were included; 128 (56.39%) had delayed presentation. The median age at diagnosis was 5 (2–4015) days, and 155 (55.51%) were male. Patients with delayed diagnosis had fewer comorbidities (50.83% vs 71.13%, *p* = 0.002) and fewer cardiac abnormalities (24.17% vs. 43.30%, *p* = 0.004). They also underwent first surgery later (median 9 (1–4170) days; *p* = 0.005). Among patients with rectoperineal fistulae, 74.51% had delayed diagnosis, compared to 42.86% of males with recto-bladder neck fistulae and 30.77% of females with cloacae (*p* = 0.38) (*p* = 0.002). Surgical complications were more frequent in the delayed group (*p* = 0.027), and 87% underwent stoma fashioning.

**Conclusion:**

Delayed ARM diagnosis is associated with increased complications and complex care. Early detection and standardised protocols are essential for improved outcomes.

## Introduction

Anorectal malformations encompass a spectrum of congenital abnormalities managed globally by paediatric surgeons. They are characterised by a lack of continuity of the anorectal canal with or without a fistula connecting parts of the perineum or urogenital tract [1, 2].

Internationally, ARM incidence ranges from 1 in 2,000 to 1 in 5,000 live births, with notable geographic disparities [3, 4]. In South Africa, the reported prevalence in the Gauteng and North West provinces combined (2.5 per 10,000 live births), including the Western Cape Province (1.79 per 10,000 live births), is consistent with global statistics [4–6]. Male predominance in ARM cases is widely documented, though some studies report higher female prevalence [6–18].

The Krickenbeck Classification categorises ARMs by anatomical features [19]. ARMs often co-occur with congenital abnormalities such as vertebral, cardiac, tracheoesophageal and genitourinary defects, influencing management and outcomes [2, 7–9, 11–17]. Neonatal ARM diagnosis relies on a thorough perineal examination, yet missed diagnoses persist in low- and middle-income as well as high-income countries [3, 10, 13, 20, 21].

Delayed diagnosis of ARM is associated with severe complications such as intestinal obstruction, perforation and increased mortality rates, particularly in premature infants [1, 6, 11, 14, 17]. Delayed diagnosis rates range substantially across countries, emphasising socio-economic and healthcare access factors [3, 7, 10, 11, 16, 17, 21–24].

Addressing delays in diagnosis requires improved neonatal examination protocols and enhanced awareness among healthcare providers and caregivers, particularly among female patients with subtle rectovestibular fistulae [10, 17]. Early detection is crucial to minimising morbidity and optimising long-term outcomes for patients with ARMs globally.

Limited literature exists on the delayed presentation of ARM in the South African context. This study aimed to retrospectively compare the profiles and outcomes of patients with ARMs and delayed presentation of ARMs at the JPCC at Chris Hani Baragwanath Academic Hospital (CHBAH) from 1 January 2018 to 31 December 2023.

## Methodology

A retrospective descriptive research design was followed. Approval to conduct the study was obtained from the Human Research Ethics Committee–Medical (M240346) of the University of the Witwatersrand.

The study was conducted in the JPCC of the Department of Paediatric Surgery at CHBAH. CHBAH is a 2680-bed central hospital in Soweto. The JPCC manages 269 patients with ARM and performs 25 to 30 posterior sagittal anorectoplasties (PSARPs) per annum.

All patients presenting with ARM to the JPCC encompassed the study population. Patients were excluded from the study when the time of ARM presentation was unknown. The sample size was determined by the number of patients with ARM during the study period and consecutive convenience sampling was used.

Data were collected electronically using REDCap by one author (AP). The data collected included the following variables: demographics **(**sex**,** birth weight, birth facility, referring province, referral level), socio-economic background (caregiver’s type of house and toilet), prenatal screening and possible abnormalities detected on ultrasound, age of delayed presentation, subtype of ARM, associated comorbidities, time from birth to first surgery and subsequent PSARP and complications.

Data were collected from the JPCC database. A patient was defined as any patient with an ARM attending the JPCC and delayed presentation as any patient with an ARM diagnosed after 24 h of birth. Bowel distension, an intraoperative finding, was defined as a complication.

Data were analysed with a biostatistician using STATA version 18.0 (StataCorp, USA). Categorical data were summarised using frequencies and percentages. Continuous variables normally distributed were summarised using means and standard deviations, and medians and interquartile ranges were used for those that were not. Data were analysed using appropriate statistical tests, including the Chi-squared test, Fisher’s exact test, and ANOVA, with a *p* value of ≤ 0.05 considered statistically significant.

## Results

Of the 269 ARM patients attending the JPCC, 42 (15.6%) of the patient’s presentation was unknown and excluded from the study. Of the included patients, 99 (43.6%) had a timely diagnosis and 128 (56.38%) had a delayed diagnosis. Of the patients with a delayed diagnosis, 66 (51.56%) were discharged home before the diagnosis was made, 5 (3.91%) were still admitted and for 57 (44.53%) patients, the information was unknown. The median age at diagnosis for the delayed group was 5 (2–4015) days.

There were no statistically significant differences between the two groups in terms of birth weight, sex, socio-economic indicators (housing and type of toilet), or referral level. The majority of patients lived in formal housing (74.7%), had access to a flushing toilet (83.2%), and underwent prenatal screening (90.0%). The method of prenatal screening did not differ significantly between groups. The patient’s demographics and referral data are shown in Table 1.

**Table 1:** Patient's demographics and referral data

Variable	Total	Timely ARM	Delayed ARM	
	Mean $\pm$ SD	Mean $\pm$ SD	Mean $\pm$ SD	*p* value
Birth weight (grams)	(*n* = 204)	(*n* = 89)	(*n* = 115)	0.838
	2879 $\pm$ 576	2883 $\pm$ 622	2900 $\pm$ 531.5	
	*n* (%)	*n* (%)	*n* (%)	*p* value
Sex	(*n* = 227)	(*n* = 99)	(*n* = 128)	
Male	126 (55.51)	58 (58.59)	68 (53.12)	0.412
Female	101 (44.49)	41 (41.41)	60 (46.88)	
Socio-economic status				
Housing	(*n* = 225)	(*n* = 98)	(*n* = 127)	
Formal house	168 (74.67)	77 (78.57)	91 (71.65)	0.237
Informal house	57 (25.33)	21 (21.43)	36 (28.35)	
Type of toilet	(*n* = 226)	(*n* = 99)	(*n* = 127)	
Inside toilet	108 (47.79)	50 (50.51)	58 (45.67)	0.650
Outside flushing toilet	80 (35.40)	35 (35.35)	45 (35.43)	
Outside pit toilet	36 (15.93)	14 (14.14)	22 (17.33)	
Community toilet	2 (0.88)	0	2 (1.57)	
Referral level	(*n* = 206)	(*n* = 88)	(*n* = 118)	
Inborn	45 (21.84)	20 (22.73)	25 (21.19)	0.210
Community health centre	38 (18.45)	12 (13.64)	26 (22.03)	
District hospital	32 (15.53)	12 (13.64)	20 (16.95)	
Regional hospital	55 (26.70)	23 (26.13)	32 (27.12)	
Tertiary and Quaternary hospital	25 (12.14)	13 (14.77)	12 (10.17)	
Private	11 (5.34)	8 (9.09)	3 (2.54)	
Prenatal screening	(*n* = 186)	(*n* = 82)	(*n* = 104)	
Yes	173 (93.01)	77 (93.90)	96 (92.31)	0.672
No	13 (6.99)	5 (6.10)	8 (7.69)	
Type	(*n* = 169)	(*n* = 75)	(*n* = 94)	
Ultrasound	15 (8.88)	6 (8.0)	9 (9.57)	0.978
Bloods	21 (12.43)	9 (12.0)	12 (12.77)	
Both	110 (65.09)	50 (66.67)	60 (63.83)	
Performed but unknown method	23 (13.60)	10 (13.33)	13 (13.83)	

There was a significant difference in the distribution of ARM subtypes between groups (*p* = 0.021). Perineal fistulae were significantly more common in the delayed diagnosis group (31.7% vs. 14.6%, *p* = 0.005). Cloacae were more frequent in the timely diagnosis group (20.2% vs. 6.7%, *p* = 0.005). Patients with delayed diagnosis had significantly fewer associated comorbidities overall (50.8% vs. 71.1%, *p* = 0.002), particularly cardiac anomalies (24.2% vs. 43.3%, *p* = 0.003). The delayed diagnosis group also had significantly fewer patients with two or more associated comorbidities (*p* = 0.011). The ARM subtypes and comorbidities are shown in Table 2.

**Table 2:** ARM subtypes and comorbidities

Variable	Total	Timely ARM	Delayed ARM	*P* value
	*n* (%)	*n* (%)	*n* (%)	
ARM subtypes	(n = 209)	(*n* = 89)	(*n* = 120)	
Major clinical groups				0.021
Vestibular	54 (25.84)	20 (22.47)	34 (28.34)	0.339
Perineal	51 (24.40)	13 (14.61)	38 (31.67)	0.005
Urethral (unknown subtype)	2 (0.96)	1 (1.12)	1 (0.83)	1
Bulbar	25 (11.96)	12 (13.48)	13 (10.83)	0.66
Prostatic	14 (6.69)	7 (7.87)	7 (5.83)	0.686
Bladder neck	7 (3.35)	4 (4.50)	3 (2.50)	0.460
Cloaca	26 (12.44)	18 (20.22)	8 (6.67)	0.005
No fistula	20 (9.57)	10 (11.24)	10 (8.33)	0.487
Anal stenosis	2 (0.96)	0	2 (1.67)	0.508
Rare/regional variants				
Rectal atresia/stenosis	4 (1.91)	1 (1.12)	3 (2.50)	0.638
Vaginal	3 (1.44)	2 (2.25)	1 (0.83)	0.576
Other	1 (0.48)	1 (1.12)	0	0.426
Comorbidities	(*n* = 217)	(*n* = 97)	(*n* = 120)	
No	87 (40.09)	28 (28.87)	59 (49.17)	0.002
Yes	130 (59.91)	69 (71.13)	61 (50.83)	
Type				
Cardiac	71 (32.72)	42 (43.30)	29 (24.17)	0.003
Renal	49 (22.58)	20 (20.62)	29 (24.17)	0.534
Vertebral	26 (11.98)	16 (16.49)	10 (8.33)	0.066
Limb	14 (6.45)	10 (10.31)	4 (3.33)	0.051
Tracheoesophageal	3 (1.38)	1 (1.03)	2 (1.67)	0.690
Other	29 (13.36)	18 (18.56)	11 (9.17)	0.047
Number of comorbidities				0.011
1	83 (38.25)	41 (42.71)	42 (35.00)	0.273
2	42 (15.20)	19 (19.79)	14 (11.67)	0.048
3	13 (5.99)	8 (8.33)	5 (4.17)	0.255
4	1 (0.46)	1 (1.04)	0	0.447

Patients with delayed diagnosis had a significantly longer time to first surgery (median 9 days, IQR 1–4170) compared to those diagnosed in a timely manner (median 2 days, IQR 0–305) *p* = 0.005). There was no significant difference in time to PSARP. Type of first surgery did not differ significantly. The timing and type of surgery are shown in Table 3.

**Table 3:** Timing and type of surgery

Variable	Total	Timely ARM	Delayed ARM	*p* value
	Median (range) (IQR)	Median (range) (IQR)	Median (range) (IQR)	
Surgery (days)				
Time from birth to first surgery	(*n* = 212)	(*n* = 93)	(*n* = 119)	
	3(0 – 4170) (20.5)	2 (0 – 305) (2)	9 (1 – 4170) (79)	0.005
Time from birth to PSARP	(*n* = 189)	(*n* = 71)	(*n* = 93)	
	165 (14–2855) (150.3)	164 (52 – 1441) (184)	166 (14 – 2855) (138.5)	0.139
	*n* (%)	*n* (%)	*n* (%)	
Type of first surgery	(*n* = 221)	(*n* = 98)	(*n* = 123)	
Stoma	190 (85.97)	83 (84.69)	107(86.99)	0.625
Primary PSARP	31 (14.03)	15 (15.31)	16 (13.01)	

Postoperative complications occurred in 83/195 (42.56%) patients following the first surgery: 70/168 (41.67%) patients that underwent stoma fashioning developed a complication, while 13/27 (48%) patients that underwent primary PSARP had complications (*p* = 0.527). Among patients with delayed diagnosis: 54 (49.54%) developed complications, with complications being more common in patients that underwent primary PSARP compared to stoma formation. Notably, marked bowel distension was the most common intraoperative finding during stoma formation (18/46 (39.1%) vs. 3/24 (12.5%), *p* = 0.028). Among patients diagnosed in a timely manner, complications were more frequently managed conservatively (12/24 (50.0%) vs. 9/46 (19.6%), *p* = 0.013), with wound dehiscence and sepsis being the most common issues. There was no significant difference in the need for surgical management between the two groups (*p* = 0.784). The complications of surgery are shown in Table 4.

**Table 4:** Complications of surgery

Variable	Total	Timely ARM	Delayed ARM	*p* value
	*n* (%)	*n* (%)	*n* (%)	
First surgery	(*n* = 195)	(*n* = 86)	(*n* = 109)	
Stoma	168 (86.15)	72 (83.72)	96 (88.07)	
Complications				
No	98 (58.33)	48 (66.67)	50 (52.08)	0.046
Yes	70 (41.67)	24 (33.33)	46 (47.92)	
Intraoperative finding stoma fashioning	30 (42.86)	5 (20.83)	25 (54.35)	0.011
Bowel distension	21 (30.0)	3 (12.50)	18 (39.14)	0.028
Bowel perforation	6 (8.57)	2 (8.33)	4 (8.70)	1
Iatrogenic bowel perforation	1 (1.43)	0	1 (2.17)	1
Bladder injury	1 (1.43)	0	1 (2.17)	1
Bladder rupture	1 (1.43)	0	1 (2.17)	1
Conservative	21 (30.0)	12 (50)	9 (19.57)	0.013
Wound dehiscence and sepsis	19 (27.14)	11 (45.83)	8 (17.40)	0.023
Strictured stoma	1 (1.43)	1 (4.17)	0	0.343
Ischaemic colon and stoma	1 (1.43)	0	1 (2.17)	1
Surgery	19 (27.14)	7 (29.17)	12 (26.09)	0.784
Bowel obstruction	4 (5.71)	1 (4.17)	3 (6.52)	1
Strictured mucous fistula	4 (5.71)	2 (8.33)	2 (4.35)	0.603
Necrotic stoma	4 (5.71)	1 (4.17)	3 (6.52)	1
Evisceration	3 (4.30)	2 (8.33)	1 (2.17)	0.269
Stoma retraction	2 (2.86)	0	2 (4.35)	0.543
Inverted stomas and wound sepsis	1 (1.43)	0	1 (2.17)	1
Unknown	1 (1.43)	1 (4.17)	0	0.343
Primary PSARP	27 (13.85)	14 (16.28)	13 (11.93)	
Complications				
No	14 (51.85)	9 (64.29)	5 (38.46)	0.473
Yes	13 (48.15)	5 (35.71)	8 (61.54)	
Conservative	5 (38.46)	1 (20.0)	4 (50.0)	0.565
Wound dehiscence	5 (38.46)	1 (20.0)	4 (50.0)	0.565
Surgery	8 (61.54)	4 (80.0)	4 (50.0)	0.565
Wound dehiscence	4 (30.78)	1 (20.0)	3 (37.50)	1
Bowel perforation	1 (7.69)	1 (20.0)	0	0.385
Missed presacral mass	1 (7.69)	1 (20.0)	0	0.385
Misplaced anus	1 (7.69)	0	1 (12.50)	1 0.385
Rectal prolapse	1 (7.69)	1 (20.0)	0	

## Discussion

Delayed diagnosis of ARM remains a substantial global problem. Delayed diagnosis has been defined in various ways by different authors, including identification after 24 h, 48 h, 7 days postpartum or after hospital discharge, beyond 3 months of age in females or when the neonate was allowed to feed without a diagnosis of ARM being established [7, 22]. In this study, 56.38% of patients presented with a delayed diagnosis. This is higher than the reported delayed diagnosis in India (43–48%) [22, 23], the United Kingdom (42–53%) [13, 24], Australia (32–38%) [3], Ireland (21.3%) [18], Egypt (20%) [21] and Canada (13%) [15] and lower than in Uganda (63.38%) [17] and Nigeria (84.6%) [11]. The delayed diagnosis of ARM is similar to Tanzania (58.7%) [16] and at the high end of the range reported in South Africa: 26% and 27% in the Western Cape [6, 7] and 57.9% in KwaZulu-Natal [10]. The elevated rate of delayed diagnosis observed at the JPCC may be partly explained by the more stringent definition applied in this study (more than 24 h), which could contribute to the difference when compared with other published data. However, the majority of patients with delayed diagnosis in our study were already discharged home from the hospital. This finding is worrying and confirms the need for standardised newborn assessment practices and training of healthcare personnel.

Consistent with existing literature, which reports a male predominance in ARM cases ranging from 56 to 68% [6–15, 21], the majority of patients in our cohort were male (55.51%). Although not statistically significant, a greater proportion of females presented with a delayed diagnosis compared to those diagnosed in a timely manner. This trend may be related to the type of fistula (rectovestibular and rectoperineal) more commonly observed in females and its associated clinical presentation.

The most common ARM subtype observed in this cohort was the rectovestibular fistula, followed by the rectoperineal fistula. Among males and females with rectoperineal fistulae, 74.51% had a delayed diagnosis, compared to 42.86% of males with recto-bladder neck fistulae and 30.77% of females with cloacae. Notably, 63% of females with a rectovestibular fistula had a delayed diagnosis. Delayed presentation in females may be due to faecal discharge through the perineal or vestibular fistula, which allows for partial decompression of the gastrointestinal tract and can obscure clinical signs. These malformations can be subtle and easily overlooked during initial examination. In contrast, males with rectourethral fistulae more often develop progressive abdominal distension, prompting earlier clinical attention [1].

In this study, the largest proportion of patients with delayed diagnosis (27.12%) were referred from regional hospitals. Interestingly, a similar percentage of patients were referred from community health centres (22.03%) as those inborn at CHBAH (21.19%). This finding is concerning, as community health centres typically operate with fewer resources and less specialised training, which may explain their contribution to delayed diagnoses. However, the fact that a comparable number of delayed cases originated from CHBAH itself, despite the availability of specialist services, suggests potential gaps in neonatal examination protocols or clinical awareness. These results highlight the need to strengthen newborn assessment practices within the hospital and improve training and diagnostic vigilance among healthcare providers, at CHBAH and in referring facilities.

Among patients with ARM, 82% had documented evidence of having undergone prenatal screening, while the remainder had confirmed records of no screening. Of those screened, 93% underwent an ultrasound, blood tests or both. Despite this, associated abnormalities were identified in only three patients, all from the timely diagnosis group, with all abnormalities involving the renal system on ultrasound. Govender et al. [10] identified that while 34% of their ARM patients had associated congenital anomalies, only 1.2% were detected prenatally. The low detection rate of comorbidities during routine prenatal screening suggests missed opportunities for earlier identification of patients with ARM, which may contribute to diagnostic delays.

Associated comorbidities were identified in 59.91% of ARM patients, consistent with the literature (61–69%) [1, 6, 8, 11, 12]. In the present study, patients with a delayed presentation exhibited significantly fewer associated anomalies compared to those diagnosed in a timely manner. Cardiac abnormalities were the most common associated condition among patients diagnosed timely, occurring in 43.30% of cases, while only 24.17% of those with a delayed diagnosis had cardiac involvement. These findings suggest that additional congenital abnormalities, particularly cardiac defects, may prompt more thorough clinical evaluation and earlier identification of ARM. This observation is consistent with findings by Theron et al. [6], who similarly reported that patients with other anomalies were more likely to be diagnosed earlier.

The authors assumed that patients from a lower socio-economic background would present delayed. However, no statistically significant difference was observed in socio-economic status between patients with delayed and timely diagnoses.

As anticipated, patients with a delayed presentation underwent their initial surgery significantly later, compared to those diagnosed in a timely manner. Among the delayed presentation group, 49.54% developed complications, notably higher than the 33.72% observed in the timely diagnosis group. Patients with delayed diagnosis were more likely to experience complications, particularly bowel distension identified during the initial stoma formation. In this study, bowel distension was classified as a complication due to its clinical implications. Although not always associated with further adverse outcomes, bowel distension can lead to abdominal distension, diaphragmatic splinting, increased risk of bowel perforation, and a more complex intra- and postoperative course potentially requiring intensive care unit admission.

While complications occurred in both groups, those diagnosed timely were more frequently managed conservatively with wound care and supportive measures. This finding underscores the importance of early recognition, as early management can prevent more severe complications and reduce the need for further procedures.

Bowel perforation was observed in six patients, of whom 83.33% had a delayed diagnosis. This is particularly concerning, given that bowel perforation following delayed presentation is associated with mortality rates as high as 30.2% and up to 50% in premature neonates with additional comorbidities [11, 14].

Consequently, decompression through colostomy formation is often required, committing both the patient and their family to further surgical procedures. Colostomy complications are not uncommon and require continuous care, impact on the psychological state of the patient and their guardians and could be avoided if patients presented promptly [21]. Although not statistically significant, a greater percentage of patients from the delayed diagnosis group underwent stoma placement compared to those in the timely diagnosis group.

An important aspect of management to consider is the selection of the surgical approach, specifically, the decision between performing a primary PSARP versus a staged repair beginning with a colostomy. While primary PSARP may be appropriate in selected cases, we have found that an initial colostomy remains the safest option in our setting [25]. This is especially relevant in the context of delayed diagnosis, where the increased risk of bowel distension, perforation, and sepsis makes a primary repair considerably more hazardous. As such, a staged approach is routinely preferred to optimise surgical outcomes and patient safety.

## Conclusion

Delayed diagnosis of ARM remains a significant challenge, with more than half of the patients in this study presenting after the ideal time frame for early intervention. This delay contributes to a range of complications, including an increased risk of bowel distension, perforation, and a more complicated postoperative course, which may necessitate additional surgeries and prolonged hospital stays. While early prenatal screening and improved neonatal assessments are critical in identifying ARM, missed opportunities for early diagnosis persist, particularly in settings with limited resources or lower clinical awareness. The findings from this study highlight the need for enhanced training and protocols in both primary healthcare settings and tertiary hospitals to ensure timely diagnosis and to reduce the risk of associated complications. By improving early detection practices, the clinical outcomes and the psychological well-being of affected families can be significantly improved. It is crucial to continue emphasising the importance of early recognition, referral, and intervention to mitigate the long-term impact of delayed ARM diagnosis.

## Limitations

The study was performed at CHBAH; therefore, the results may not be generalisable to other hospitals. However, this study has provided important information regarding patients treated at CHBAH.

## Data Availability

No datasets were generated or analysed during the current study.
